# Age-Associated Weight Gain, Leptin, and SIRT1: A Possible Role for Hypothalamic SIRT1 in the Prevention of Weight Gain and Aging through Modulation of Leptin Sensitivity

**DOI:** 10.3389/fendo.2015.00109

**Published:** 2015-07-16

**Authors:** Tsutomu Sasaki

**Affiliations:** ^1^Laboratory for Metabolic Signaling, Institute for Molecular and Cellular Regulation, Gunma University, Maebashi, Japan

**Keywords:** aging, energy homeostasis, energy sensing, inflammation, insulin resistance, leptin resistance, sirtuin, ubiquitin-proteasome system

## Abstract

The hypothalamus is the principal regulator of body weight and energy balance. It modulates both energy intake and energy expenditure by sensing the energy status of the body through neural inputs from the periphery as well as direct humoral inputs. Leptin, an adipokine, is one of the humoral factors responsible for alerting the hypothalamus that enough energy is stored in the periphery. Plasma leptin levels are positively linked to adiposity; leptin suppress energy intake and stimulates energy expenditure. However, prolonged increases in plasma leptin levels due to obesity cause leptin resistance, affecting both leptin access to hypothalamic neurons and leptin signal transduction within hypothalamic neurons. Decreased sensing of peripheral energy status through leptin may lead to a positive energy balance and gradual gains in weight and adiposity, further worsening leptin resistance. Leptin resistance, increased adiposity, and weight gain are all associated with aging in both humans and animals. Central insulin resistance is associated with similar observations. Therefore, improving the action of humoral factors in the hypothalamus may prevent gradual weight gain, especially during middle age. SIRT1 is a NAD^+^-dependent protein deacetylase with numerous substrates, including histones, transcription factors, co-factors, and various enzymes. SIRT1 improves both leptin sensitivity and insulin sensitivity by decreasing the levels of several molecules that impair leptin and insulin signal transduction. SIRT1 and NAD^+^ levels decrease with age in the hypothalamus; increased hypothalamic SIRT1 levels prevent age-associated weight gain and improve leptin sensitivity in mice. Therefore, preventing the age-dependent loss of SIRT1 function in the hypothalamus could improve the action of humoral factors in the hypothalamus as well as central regulation of energy balance.

## Introduction

In this review, I will first highlight the impact of obesity prevalence and the importance of age-associated weight gain in this context. Next, I will provide an overview of the central mechanisms for regulating body weight, including a discussion of central leptin/insulin resistance during aging and obesity. Finally, I will explore how hypothalamic SIRT1 may be involved in age-associated weight gain and diet-induced obesity by regulating leptin and insulin sensitivity in the central nervous system. I propose that hypothalamic SIRT1 dysfunction could be a cause of weight gain associated with aging and diet.

## Underestimated Prevalence of Obesity-Associated Health Issues

Obesity is a worldwide health concern. Based on the World Health Organization’s definition of obesity [body mass index (BMI) >30 kg/m^2^], more than 600 million adults were obese and more than 1.9 billion adults were overweight (BMI 25–30 kg/m^2^) in 2014 (1). In other words, ~13% of the world’s adult population (11% of men and 15% of women) was obese in 2014. These numbers are alarming, but they underestimate the actual impact on health care caused by obesity throughout the world.

**Figure 1:**
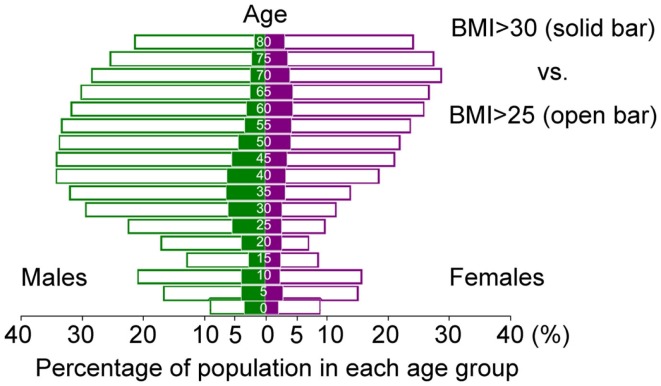
**Age-dependent distribution of Japanese with BMI > 30 kg/m^2^ (solid bar) and BMI > 25 kg/m^2^ (open bar) in 2014**. (Based on data from the Institute for Health Metrics and Evaluation (IHME). Overweight and Obesity Viz. Seattle, WA, USA: IHME, University of Washington, 2014. Available from: http://vizhub.healthdata.org/obesity).

Ethnic groups exhibit differing susceptibilities to obesity-associated diseases, such as diabetes. In particular, Asians are more vulnerable to the detrimental effects of obesity than Caucasians. The prevalence of diabetes among Asian descendants living in the United States and Canada with BMI of 25 kg/m^2^ is equal to that of Caucasians with BMI of 30 kg/m^2^ (2, 3). This phenomenon is partly due to different pancreatic beta-cell reserve capacity (4), but Asians also have more visceral adipose mass than Caucasians at the same BMI (5). Based on the risk of developing metabolic syndrome, the definition of obesity in Japan is BMI > 25 kg/m^2^, not BMI > 30 kg/m^2^ (6). Although ~5% of Japanese males in their 30s and 40s are obese according to the World Health Organization’s definition (BMI > 30 kg/m^2^), this number increases to ~35% if the Japanese definition of obesity (BMI > 25 kg/m^2^) is used (Figure 1) (7). Therefore, the true number of people at health risk due to “obesity” around the world is somewhere between 600 million (BMI > 30 kg/m^2^) and 2.5 billion (BMI > 25 kg/m^2^).

Obesity is commonly discussed in the context of metabolic syndrome and other diseases that result from obesity. However, the prevalence of obesity is positively associated with age, and the global emergence of overweight and obesity is compounded by simultaneous aging of the population (8, 9). Obesity itself is a risk factor for age-associated diseases (10–13); it accelerates cellular processes in a manner similar to aging and shortens lifespan (14–17). Therefore, understanding gradual declines in quality of health through aging and obesity is as important as simply understanding the consequences of obesity.

Although age-associated weight gain is important for understanding gradual declines in quality of health, fewer studies have addressed the mechanisms of these declines than have investigated diet-induced obesity, partly because the former process is much harder to study than the latter. Although it is difficult to completely separate the contributions to weight gain of age and diet, not everyone in the world eats the high-calorie diet employed in studies of diet-induced obesity; a significant proportion of the population gains weight during middle age. Therefore, outreach strategies targeting age-associated weight gain are expected to impact the health of many people.

Do different mechanisms underpin weight gain associated with age and weight gain caused by diet, or do common mechanisms underlie both processes? In order to tackle the obesity crisis, we must understand how body weight is regulated, as well as how the intricate system that regulates energy homeostasis could be disrupted through both aging and diet.

## Brief Overview of Homeostatic Control of Body Weight and Energy Balance by the Central Nervous System

**Figure 2:**
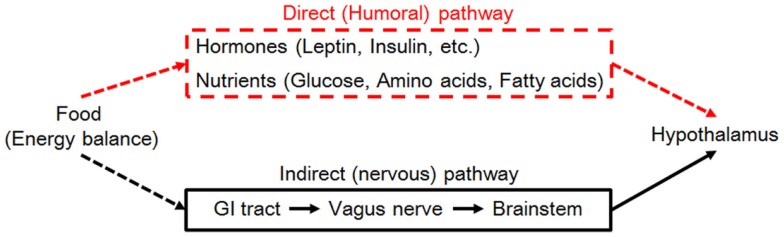
**Neural signaling (right, black lines) and hormonal signaling (left, red lines) from the periphery are required for nutrient/energy sensing by the hypothalamus (blue) to regulate body weight**. Abbreviation: GI, gastrointestinal.

The hypothalamus is the center of homeostatic control of body weight and energy balance. It integrates energy information conveyed from the periphery by nutrients and hormones through two pathways (neural and humoral) and regulates energy intake and expenditure (Figure 2).

The neural pathway senses peripheral energy status via vagal afferents innervating the gastrointestinal tract and hepatic portal veins. Nutritional information gathered by vagal afferents is sent to the solitary tract of the brainstem and subsequently into the hypothalamus. The humoral pathway consists of direct input of nutrients and hormones to the primary center for body-weight control: the arcuate nucleus of the hypothalamus (ARC). The ARC is located close to the median eminence, which provides humoral factors with access to the central nervous system. Humoral factors can enter the cerebrospinal fluid (CSF) in the third ventricle and gain access to ARC neurons because the tanycytes lining the third ventricle adjacent to the ARC are permissive to these nutrient cues (18).

**Figure 3:**
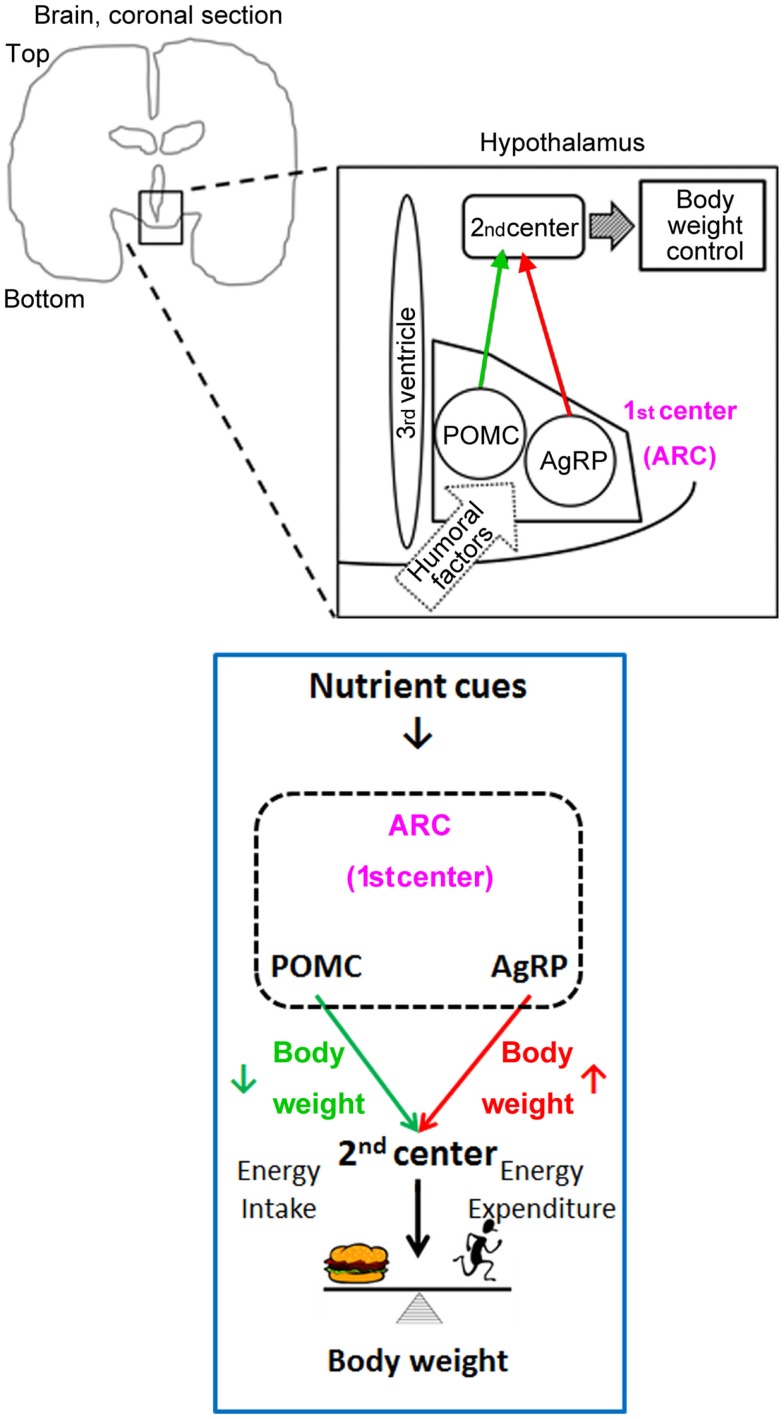
**Humoral factors leptin and insulin, that convey nutrient cues, act on the central melanocortin system (POMC and AgRP neurons) in the ARC to regulate body weight**.

Once nutrients and hormones gain access to ARC neurons, they can directly affect neuronal activity. Two major subtypes of neurons located in the ARC are proopiomelanocortin (POMC) neurons and Agouti-related peptide (AgRP) neurons; both types of neurons receive signals containing information about energy status (19). POMC neurons promote weight loss and AgRP neurons promote weight gain. These neurons provide similar projections to secondary centers for body-weight control, such as the paraventricular nucleus of the hypothalamus and the lateral hypothalamus (Figure 3). Activity of the neurons in these secondary centers may be regulated partly through the melanocortin type 4 receptors, since POMC neurons secrete the agonist α-melanocyte-stimulating hormone (made by processing the POMC peptide) and AgRP is an inverse agonist for melanocortin type 4 receptors. AgRP neurons also use the neurotransmitters neuropeptide Y and GABA to suppress target-neuron activity, including that of POMC neurons in the ARC. POMC neurons, AgRP neurons, and melanocortin type 4 receptors comprise the central melanocortin system.

Leptin is an adipokine critical to the regulation of energy homeostasis (20). Intracellular leptin signaling is initiated by leptin binding to the long form of leptin receptor (LepRb), which has the intracellular domain, causing a conformational changes in LepRb and promoting the phosphorylation and activation of Jak2 (21). Activated Jak2 phosphorylates three tyrosine residues in the cytoplasmic domain of LepRb (Y985, Y1077, and Y1138), leading to further signal transduction involving STAT3, STAT5, and the ERK and PI3K/AKT pathways. LepRb is expressed in several brain nuclei, including the ARC, the paraventricular nucleus of the hypothalamus, and the dorsomedial, lateral, and ventromedial regions of the hypothalamus (22). Mutations in leptin, leptin receptor, and the central melanocortin system are the most common mutations in the monogenic form of obesity in humans (23). A homozygous loss-of-function mutation in the leptin receptor (the *db* mutation) causes hyperphagia and obesity, leading to diabetes in mice (24, 25).

Insulin is a pancreatic hormone that plays a crucial role in lowering blood glucose levels. It also contributes to the regulation of body weight. Although insulin acts on peripheral tissues (such as liver, skeletal muscle, and adipose tissue) to cause anabolic effects (to cause weight gain), insulin act as a catabolic anorexigenic signal in the central nervous system (to cause weight loss) (26). The insulin receptor is expressed widely in the central nervous system (27). Insulin binding causes the insulin receptor to recruit insulin receptor substrate (IRS) proteins. IRS-2 is the main mediator of insulin intracellular responses in the brain (28, 29). IRS protein activation leads to activation of PI3K and subsequent activation of AKT. Although the glucose-lowering effect of insulin is mediated by the insulin-sensitive glucose transporter GLUT4 in the periphery, glucose transport into most neurons is GLUT3-dependent; transport into the glia and brain endothelial cells is GLUT1-dependent (30). Therefore, insulin is not needed for glucose transport into most brain cells. Instead, insulin in the brain is important for the central regulation of energy homeostasis and glucose homeostasis.

Both leptin and insulin regulate POMC neurons and AgRP neurons at multiple levels. They regulate the transcription of *Pomc* and *AgRP* via their downstream target transcription factors STAT3 and FoxO1, respectively, and promote the expression of anorexigenic POMC while suppressing orexigenic AgRP (31, 32). FoxO1 also regulates the expression of carboxypeptidase E, which is necessary for processing POMC into α-melanocyte-stimulating hormone (an agonist of melanocortin type 4 receptors) (33). Leptin and insulin also regulate the activities of POMC neurons and AgRP neurons (34). For example, both leptin and insulin act on POMC neurons to increase sympathetic activity to adipose tissues and to promote the browning of white fat (35). Therefore, these two hormonal signals of satiety regulate transcription, peptide processing, the activity of central melanocortin neurons, and energy and glucose homeostasis.

## Three Layers of Leptin/Insulin Resistance in the Central Regulation of Body Weight

The anorexigenic hormone leptin needs to enter the central nervous system and reach target neurons in order to suppress food intake and to stimulate energy expenditure. Leptin action encounters three barriers: the blood–CSF barrier, leptin uptake by the target neuron, and intracellular leptin-signaling resistance.

First, leptin must exit the bloodstream and gain access to the CSF. The short isoform of the leptin receptor is highly expressed in cerebral microvessels and choroid plexuses, and is considered to be the main receptor by which leptin crosses the blood–brain barrier and the blood–CSF barrier (36). Megalin expressed by choroid plexuses can also act as a receptor to transport leptin from the blood to the CSF (37). The fenestrated endothelium of median eminence microvessels and the tight junctions between tanycytes together compose the blood–CSF barrier adjacent to the ARC (18). VEGF-A expression in tanycytes modulates the properties of this barrier, and ERK signaling in tanycytes promotes leptin transport across tanycytes to the CSF (38, 39). Once leptin enters the CSF/brain, it must find its target receptor (LepRb), but gliosis can interfere with the diffusion of leptin to LepRb on the target neurons (40). Finally, intracellular leptin signaling can be down-regulated by several molecules, such as PTP-1B, TC-PTP, SOCS3, and endospanin 1, which act on JAK2, STAT3, LepRb, and LepRb endocytosis, respectively (41, 42).

**Figure 4:**
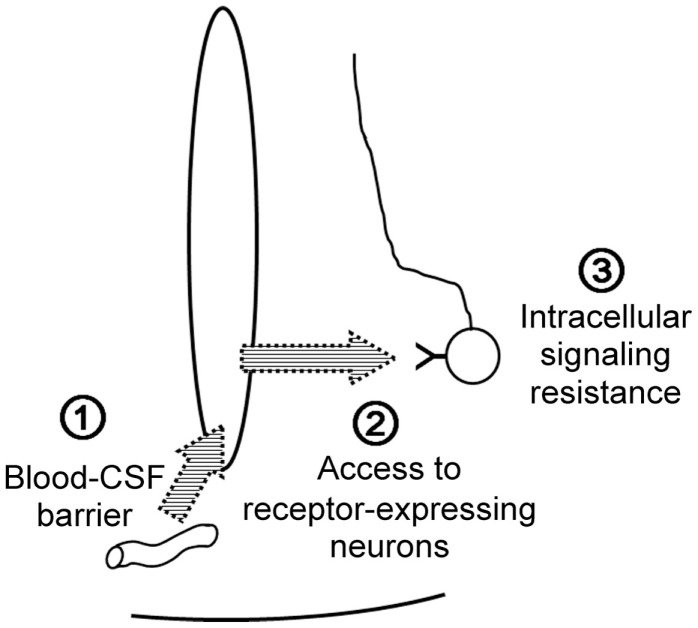
**Common mechanisms for central leptin/insulin resistance**.

Although the source of central insulin is debated, peripheral administration of insulin raises insulin concentrations within the CSF (43), indicating that peripheral insulin also serves as a satiety signal for the central nervous system by crossing the blood–brain barrier (44). Kinetic studies indicate that plasma-to-CSF transport of insulin involves a saturable mechanism (45, 46); this transport is decreased in several insulin-resistant states (47–49). Megalin is implicated in insulin transport, at least across renal tubular epithelial cells (50), which suggest that megalin may also work at the blood–CSF barrier as a carrier for insulin. Both PTP-1B and SOCS3 have been reported to cause insulin resistance at the level of the insulin receptor and IRS proteins, respectively (26). Therefore, common mechanisms are involved in central resistance to leptin and insulin (Figure 4).

## Diet-Induced Obesity and Aging Cause Central Resistance to Leptin and Insulin

Diet-induced obesity causes central leptin resistance first by affecting the central access of leptin and later by causing leptin resistance within the central nervous system (51). Obesity is associated with decreased leptin transport across the blood–brain barrier in rats (36) and in humans (52), which explains why obese humans have low CSF leptin levels despite having high-serum leptin levels. Within the central nervous system, diet-induced obesity does not uniformly cause leptin resistance. Diet-induced obesity caused by intake of a chronic high-fat diet mainly induces cellular leptin resistance in the ARC and the ventral tegmental area, but not in the lateral hypothalamus, ventromedial hypothalamus, and dorsomedial hypothalamus (53). Diet-induced obesity also induces astrogliosis (along with activation of microglia), which prevents circulating metabolic feedback factors, such as leptin from accessing neurons (54). Aging also impairs the central response to leptin (55–59) by impairing central leptin access and cellular leptin signaling (60). Aging is associated with down-regulation of megalin expression (37), decreased leptin uptake in the hypothalamus due to decreased expression of leptin receptor mRNA (61), decreased levels of leptin receptor protein in the hypothalamus (62), and increased PTP1B levels in the hypothalamus (63).

The central insulin response is also attenuated by aging and diet-induced obesity (64–67). Insulin levels in the CSF are paradoxically low- compared to high-serum insulin levels in obese humans (68) and in genetically induced and diet-induced obese animals (49, 69). Consumption of a high-fat diet triggers hypothalamic angiopathy (70); endothelial cells of brain microvessels in obese fa/fa rats exhibit reduced insulin binding, leading to reduced internalization of the insulin–insulin receptor complex in rat brain endothelial cells (71). Expression of the insulin receptor decreases with aging in the central nervous system, especially in the hypothalamus, cerebral cortex, and hippocampus in rats (72). Central administration of insulin fails to reduce food intake under conditions of obesity (65, 67, 73), and intranasal application of insulin to the human brain improves peripheral insulin sensitivity in lean but not in obese men (74), indicating that obesity also causes central insulin resistance.

Therefore, previous studies indicate that both central leptin resistance and central insulin resistance are induced by diet-induced obesity and aging at multiple levels. Does a common mechanism underlie these phenomena?

## Hypothalamic Inflammation as a Culprit for Central Leptin/Insulin Resistance

**Figure 5:**
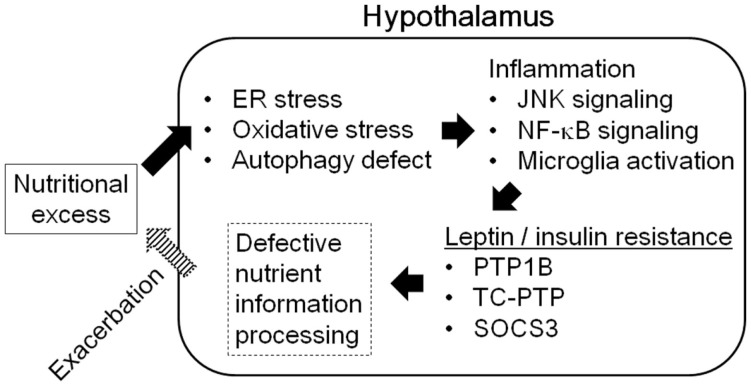
**Hypothalamic inflammation and central leptin/insulin resistance**.

Hypothalamic inflammation is induced during diet-induced obesity both in rodents and in humans (75). Although intracellular fatty-acid sensing within the hypothalamus is important for the regulation of energy balance (76), excessive amounts of fatty acid in the diet cause hypothalamic inflammation and lead to obesity. Consumption of a diet rich in saturated fatty acids promotes inflammation, gliosis, and neuronal stresses in the mediobasal hypothalamus, along with inflammatory activation of microglia. Depleting microglia from the mediobasal hypothalamus of mice blocks the inflammation and neuronal stresses induced by dietary saturated fatty acids and improve leptin sensitivity (77). Saturated fatty acids can activate toll-like receptor 2- and 4-dependent signaling, leading to induction of the pro-inflammatory signaling mediated by JNK and NF-κB (78, 79). Excess amounts of free fatty acids, glucose, and amino acids due to over-nutrition induce endoplasmic reticulum (ER) stress and oxidative stress, which also promote the activation of pro-inflammatory signaling and defective autophagy (79). These metabolically induced pro-inflammatory changes in the hypothalamus cause defective intracellular leptin and insulin signaling, leading to central leptin/insulin resistance (80–84) (Figure 5).

Pro-inflammatory signals in the hypothalamus are also involved in the aging of mice. Aging activates microglia and NF-κB signaling in the hypothalamus, inhibiting these reactions can improve age-dependent declines in body function in mice (85). Therefore, hypothalamic inflammation, specifically microglial activation and induction of NF-κB signaling, is involved in age-dependent and diet-induced obesity, which are accompanied by central leptin/insulin resistance.

## Hypothalamic SIRT1 Ameliorates Central Leptin Resistance and Central Insulin Resistance

SIRT1 is an NAD^+^-dependent protein deacetylase (86) and an energy-sensing molecule responsible for promoting healthy longevity through caloric restriction (87). Caloric restriction prevents aging-associated central leptin resistance in rats (88, 89). In peripheral tissues, SIRT1 has been demonstrated to promote fatty-acid oxidation and to improve insulin sensitivity (90). Single nucleotide polymorphisms in human *SIRT1* are linked to both adult obesity (91–94) and childhood obesity (95), indicating that SIRT1 may regulate body weight.

Several groups have reported genetic manipulation of SIRT1 in the hypothalamus and analyzed the effects of this manipulation on energy and glucose homeostasis. Genetic loss of *Sirt1* in anorexigenic POMC neurons causes leptin resistance and decreases energy expenditure, whereas increasing SIRT1 levels in POMC neurons improves leptin sensitivity and ameliorates the decreased energy expenditure caused by an insulin-resistant form of FOXO1 in mice (96–98). Genetic loss of *Sirt1* in orexigenic AgRP neurons causes weight loss due to decreased food intake via reductions in the firing ability of AgRP neurons in mice (99). Increasing SIRT1 levels in AgRP neurons also decreases food intake and body weight due to improved nutrient/hormone sensing in mice (97). Genetic manipulation of *Sirt1* in steroidgenic factor 1 (SF1)-positive ventromedial hypothalamic neurons alters sensitivity to leptin and orexin-A, and modulates glucose uptake by skeletal muscle via the sympathetic nervous system (100). Therefore, SIRT1 stimulates energy expenditure, suppresses food intake, and regulates glucose homeostasis in POMC neurons, AgRP neurons, and ventromedial hypothalamic SF1-positive neurons, respectively, partly through improved nutrient/hormone sensing.

How does SIRT1 improve central leptin/insulin sensitivity? SIRT1 can down-regulate proteins that promote leptin resistance, such as PTP1B, TC-PTP, and SOCS3 (97, 101). It also promotes insulin sensitivity by suppressing PTP1B [which also contributes to insulin resistance (101)], promoting IRS2 function (102), and down-regulating the transcription factor FOXO1 by promoting its degradation (98, 103). Down-regulation of NF-κB signaling may also improve central leptin/insulin sensitivity because SIRT1 suppresses inflammatory reactions by suppressing the p65RelA subunit of NF-κB (104). SIRT1 levels are reported to decrease as microglia age, and microglial SIRT1 deficiency plays a causative role in aging-mediated memory deficits in mice (105). However, the actions of microglial SIRT1 in the context of obesity, induced by aging or by diet, remain elusive. Other candidates for SIRT1’s downstream effects on central leptin/insulin sensitivity are autophagy and ER stress, since disruption of autophagy and increased ER stress are both linked to hypothalamic leptin resistance and perturbed energy homeostasis (80, 82, 83, 106, 107); SIRT1 regulates both autophagy and ER stress through substrates, such as LC3 (108), Atg5, Atg7, Atg8 (109), and XBP1s (110). However, these hypotheses need to be tested experimentally.

SIRT1 can influence epigenetic regulation by directly modifying histones (86) and by affecting DNA methylation through DNMT1 (111) and MeCP2 (112). Thus, SIRT1 can modify the global transcriptional landscape through epigenetic regulation and the targeting of numerous transcription factors and co-factors (90). Therefore, unidentified molecules may also play roles in the improvement of central leptin/insulin sensitivity by SIRT1.

## Potential Role of Hypothalamic SIRT1 in Weight Gain and Aging

Overall, SIRT1 in the hypothalamus improves energy and glucose homeostasis and central leptin/insulin sensitivity. Because there is no single reliable marker for SIRT1 function *in vivo*, here I discuss changes in NAD^+^ content and SIRT1 protein levels during aging and diet-induced obesity in the hypothalamus.

NAD^+^ content in the brain decreases with age in humans and in mice (113, 114). In mice, hypothalamic NAD^+^ content is significantly decreased in diet-induced obesity (after 4 weeks of a high-fat, high-sucrose diet) and in genetically induced obesity (with a homozygous *db* mutation) (97). Nicotinamide phosphoribosyltransferase (NAMPT), an essential enzyme in the NAD^+^ biosynthetic pathway that converts nicotinamide into nicotinamide mononucleotide, exists in two forms (intracellular and extracellular) in mammals (115). The brain expresses intracellular NAMPT at very low levels, and NAMPT levels decrease with aging in multiple organs (116, 117). The extracellular form of NAMPT is secreted from adipose tissue to affect NAD^+^ concentrations in the hypothalamus. Systemic injection of a NAMPT-neutralizing antibody decreases hypothalamic NAD^+^ content (118). Adipose tissue-specific *Nampt* knockout mice exhibit reduced plasma levels of extracellular NAMPT as well as reduced NAD^+^ concentrations in the hypothalamus; in contrast, adipose tissue-specific *Nampt* knock-in mice display increased plasma levels of extracellular NAMPT and increased hypothalamic NAD^+^ content (118). Therefore, although the brain relies on circulating extracellular NAMPT and nicotinamide mononucleotide for NAD^+^ biosynthesis, the supplies of these precursors decrease with age. Supplementation with these precursors effectively prevents diet-induced obesity and aging-induced diabetes in mice (116, 119), and therefore, this supplementation is currently under exploration as a strategy for counteracting aging and obesity in humans.

Restoring SIRT1 protein levels is a less-explored approach to this goal. SIRT1 levels within the ARC decrease with age (97, 120) and after 4 weeks of consumption of a high-fat, high-sucrose diet in mice (97). Increasing SIRT1 protein levels specifically in POMC neurons or in AgRP neurons via genetic approaches was sufficient to prevent age-associated weight gain by stimulating energy expenditure and by suppressing food intake through improved leptin sensitivity (97). However, feeding mice a high-fat, high-sucrose diet reduced both endogenous SIRT1 protein and overexpressed SIRT1 protein in ARC, and eliminated the beneficial effects of genetic overexpression of SIRT1. Therefore, along with increasing SIRT1 activity by providing more NAD^+^ or a pharmacological activator, increasing the effective enzyme concentration *per se* also yields benefits. To enable such a SIRT1 “booster” approach, the mechanisms responsible for the decline in ARC SIRT1 levels during diet-induced obesity and/or aging must be identified.

Diet similarly affected ARC SIRT1 protein levels when SIRT1 was expressed from the endogenous *Sirt1* locus and when coding-sequence *Sirt1* cDNA (without any 5′-UTR or 3′-UTR) was expressed from the endogenous *Rosa26* promoter (97). These data indicate that the diet-induced reduction in ARC SIRT1 levels is unlikely to be driven by transcriptional or post-transcriptional regulation, but is likely mediated by post-translational regulation. SIRT1 degradation *in vitro* and *in vivo* could be regulated by the ubiquitin-proteasome system in the hypothalamus (121) and possibly in other tissues (122–124). Although exposure to a 4-week, high-fat, high-sucrose diet was sufficient to decrease ARC SIRT1 levels in mice, neither a high-fat diet nor a high-sucrose diet alone was as effective (unpublished observation). Furthermore, the effect was not as clearly evident after 2 weeks of a high-fat, high-sucrose diet (unpublished observation). Therefore, declines in ARC SIRT1 levels may not directly result from nutrients within the diet, but rather may be due to metabolic changes within the hypothalamus or in the periphery caused by chronic over-nutrition. Pro-inflammatory signaling could underlie the declines in ARC SIRT1 levels during aging and during diet-induced obesity because inflammatory changes occur during aging and during chronic over-nutrition.

## Conclusion

**Figure 6:**
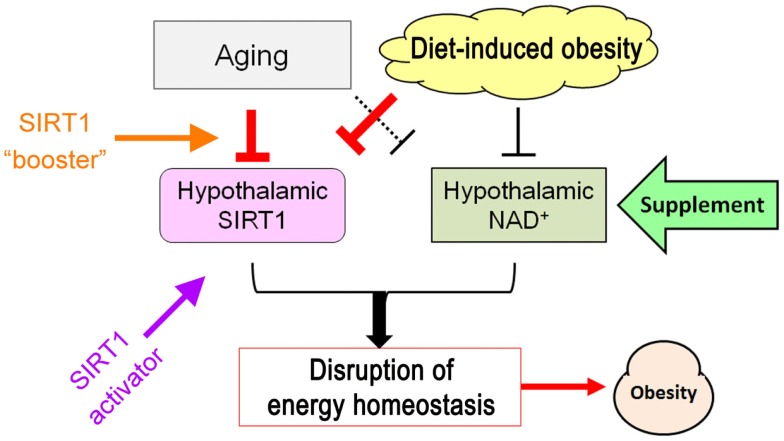
**Schematic of potential strategies to ameliorate hypothalamic SIRT1 dysfunction caused by aging- and diet-induced obesity**.

Central leptin and insulin resistance, hallmarks of disrupted nutrient/energy sensing by the hypothalamus, are common mechanisms for weight gain caused by aging and diet. Hypothalamic SIRT1 can improve these disruptions by acting on several targets that cause central leptin/insulin resistance. Meanwhile, aging and diets that promote weight gain suppress hypothalamic SIRT1 function by affecting the levels of both SIRT1 and NAD^+^, which is required for SIRT1 activity. Restoration of hypothalamic SIRT1 function can prevent age-associated weight gain in mice, indicating that improving hypothalamic SIRT1 function at multiple levels via supplementation with NAD^+^ intermediates, SIRT1 activators, and a SIRT1 “booster” may enable novel treatments of weight gain related to metabolic syndromes as well as weight gain caused by aging (Figure 6).

## Conflict of Interest Statement

The author declares that the research in this review was conducted in the absence of any commercial or financial relationships that could be construed as a potential conflict of interest.
